# Proliferation of atmospheric datasets can hinder policy making: a data blending technique offers a solution

**DOI:** 10.3389/fdata.2023.1198097

**Published:** 2023-08-08

**Authors:** Hamish Steptoe, Theo Economou

**Affiliations:** ^1^Met Office, Exeter, United Kingdom; ^2^Climate and Atmosphere Research Centre (CARE-C), The Cyprus Institute, Nicosia, Cyprus

**Keywords:** data science, decision making, data blending, precipitation, Nepal, Generalized Additive Models, statistics

## Abstract

The proliferation of atmospheric datasets is a key outcome from the continued development and advancement of our collective scientific understanding. Yet often datasets describing ostensibly identical processes or atmospheric variables provide widely varying results. As an example, we analyze several datasets representing rainfall over Nepal. We show that estimates of extreme rainfall are highly variable depending on which dataset you choose to look at. This leads to confusion and inaction from policy-focused decision makers. Scientifically, we should use datasets that sample a range of creation methodologies and prioritize the use of data science techniques that have the flexibility to incorporate these multiple sources of data. We demonstrate the use of a statistically interpretable data blending technique to help discern and communicate a consensus result, rather than imposing a priori judgment on the choice of dataset, for the benefit of policy decision making.

## 1. Introduction

Data underpins all scientific analysis. But bridging the gap from science to policy making typically requires a consistent scientific message; uncertainty or a lack of consensus is frequently used to justify inaction (Mccright and Dunlap, 2000; Orlove et al., 2020). In atmospheric science, the availability of data on which to conduct scientific analysis is considerable and reflects the wide variety of data collection and processing techniques. To demonstrate the variation of results that could be obtained from common, open access datasets, we examine summer rainfall extremes in Nepal.

In Nepal, the South Asian summer monsoon season (June to September, JJAS), contributes 70–80% of annual rainfall totals (DHM, 2022). Extreme precipitation events that occur in the monsoon season have wide-ranging impacts including flooding and landslides, which can be damaging and costly to a variety of infrastructure. Within the context of hydropower, damage to infrastructure as a result of climate induced hazards, is most often associated with extreme rainfall accumulation occurring in the monsoon season (Basnyat and Watkiss, 2017). Therefore, planning new infrastructure projects, such as hydropower plants or urban development, should incorporate an understanding of the likelihood of extreme rainfall events.

## 2. Methods

The key requirements for our data blending framework is the ability to: (i) be spatially and temporally consistent, (ii) account for data hierarchy, (iii) integrate unobserved uncertainty that accounts for the variability between datasets and (iv) flexibly characterize the variability in the data via a range of possible probability distributions. We construct our data blending framework based on Generalized Additive Models (GAMs) (Hastie and Tibshirani, 1990; Wood, 2017). For this case study, we demonstrate extreme value analysis of RX1day JJAS block-maxima of extreme precipitation, modeled using the Generalized Extreme Value (GEV) distribution:


$$\begin{array}{l} \;Y_{s,t,m}\sim GEV\left(\mu_{s,t,m},\;\sigma_{s,t,m},\;\xi_{m}\right)\\ \;\mu_{s,t,m}=\;\beta_{0}+\;ℱ\left(year_{t}\right)+\;G\left(lon_{s},lat_{s}\right)\\ \;+ℋ(lon_{s},lat_{s},\;u_{m}^{\left(\mu \right)})+\epsilon_{i}\\ \;\log(\sigma_{s,t,m})=\;\gamma_{0}+\;ℱ(year(t))+G(lon_{s},lat_{s})\\ \;+ℋ(lon_{s},lat_{s},u_{m}^{\left(\sigma \right)})+\epsilon_{i}\\ \;logit\left(\xi_{m}\right)=\delta_{0}+\;u_{m}^{\left(\xi \right)}+\epsilon_{i} \end{array}$$

where *Y*_*s, t, m*_ represents RX1day precipitation maximum for grid *s*, year *t* and dataset *m*. Following extreme value theory, *Y*_*s, t, m*_ is modeled using the GEV distribution with μ_*s, t, m*_ (location), σ_*s, t, m*_ (scale) and ξ_*m*_ (shape) parameters, that vary in space, time and dataset. The model is a HGAM (Pedersen et al., 2019) with intercepts (β_0_, γ_0_, δ_0_) and selected covariates accounting for long-term variability in time F(year(t)), variability in space $G\left(lon_{s},lat_{s}\right)$ and dataset-specific deviations, $H\left(lon_{s},lat_{s},u_{m}\right)$, that describe how each individual dataset *m* deviates from the overall spatial field G(·). Functions F(·), G(·) and H(·) are smooth functions of their respective covariates (year, longitude, latitude and dataset-specific random effects) that are estimated in the GAM fitting process. Within the GAM framework, these functions are a sum of smooth basis functions, so that for the μ_*s, t, m*_ parameter for example:


$$\begin{array}{l} ℱ\left(year_{t}\right)\;=\;\sum_{k=1}^{K}\beta_{k}\;b_{k}(year_{t})\\ \;G\left(lon_{s},lat_{s}\right)=\;\sum_{m=1}^{M}\sum_{n=1}^{N}\beta_{m,n}\;c_{n}(lat_{s})d_{m}(lon_{s})\\ ℋ\left(lon_{s},lat_{s},u_{m}\right)=\sum_{p=1}^{P}\sum_{q=1}^{Q}\sum_{r=1}^{R}\beta_{p,q,r}\;e_{r}\left(u_{m}\right)\\ \;y_{q}\left(lat_{s}\right)x_{p}\left(lon_{s}\right) \end{array}$$

where K, M and N are the number of knots that define the complexity (or wiggliness) of the basis functions *b*(·), *c*(·), *d*(·), *x*(·) and *y*(·) with corresponding model coefficients β that are specific to each GEV parameter (but not denoted to avoid notational clutter). Equivalent functions for model coefficients γ and δ are also constructed. Note that *e*(·) is a special case whereby the dataset specific random effect is treated as a smooth function (see Wood, 2006, 2008; and Wood et al., 2013 for further details) where *e*(·) is a ridge spline basis that emulates an independent and identically distributed Gaussian random effect. This adds as a constraint across data sets *m* for H(·). The R code used to fit this model is available in the Supplementary material.

Data blending is achieved via the “global” terms [the intercepts β_0_, γ_0_ and δ_0_, and functions F(·) and G(·)] which are assumed common across *m*. Dataset-specific parameters $u_{m}^{\left(\cdot \right)}$ capture variability in the data (other than that explained by the global terms) due to discrepancies in each dataset. These parameters are assumed to be random effects and are modeled via:


$$\begin{array}{lll} u_{m}^{\left(\mu \right)} & \sim & Normal\left(\mu_{\mu },\sigma_{\mu }^{2}\;\right),\\ u_{m}^{\left(\sigma \right)}\; & \sim & \;Normal\left(\mu_{\sigma },\sigma_{\sigma }^{2}\;\right),\\ u_{m}^{\left(\xi \right)}\; & \sim & \;Normal\;\left(0,\sigma_{\xi }^{2}\right). \end{array}$$

Where the parameters for these distributions are estimated from the model fitted parameters from *e*(·). Assuming these to be random quantities, explicitly captures variability from using multiple data sources, thus allowing predictions for datasets other than the ones used for estimation (e.g., radar measurements). These terms are designed to simulate within the model, additional unsampled dataset variability, as if we had incorporated more than *m* datasets. In fact, this formulation allows the uncertainty associated with different observed datasets to be integrated out for each GEV parameter e.g.,


$$\begin{array}{l} \mu_{s,t}=\;\int_{u_{s,m}^{\left(\mu \right)}}^{}\mu_{s,t,m}\;du_{m}^{\left(\mu \right)}\\ \sigma_{s,t}=\;\int_{u_{s,m}^{\left(\sigma \right)}}^{}\sigma_{s,t,m}\;du_{m}^{\left(\sigma \right)}\\ \;\xi =\;\int_{u_{m}^{\left(\xi \right)}}^{}\xi_{m}\;du_{m}^{\left(\xi \right)} \end{array}$$

which are the blended estimates of the GEV parameters. Finally, predictions of the variable of interest are based on:


$$\begin{array}{l} Y_{s,t}\sim GEV\left(\mu_{s,t},\;\sigma_{s,t},\;\xi \right). \end{array}$$

Although we illustrate the method using a GEV distribution fitted to rainfall extremes, the choice of predictive distribution, or mixture of distributions (e.g. Economou et al., 2023), should be tailored to the variable of interest. In some cases, suitable distributions may still produce unrealistic values. In this example, the GEV distribution may produce negative values for some GEV parameters, which would be unrealistic for rainfall. Identifying the most suitable predictive distribution may also be the primary limitation of this method. In some cases, it may be intractable to identify the mixture of distributions responsible for producing the data given conditional differences of the observing system.

## 3. Results

**Figure 1:**
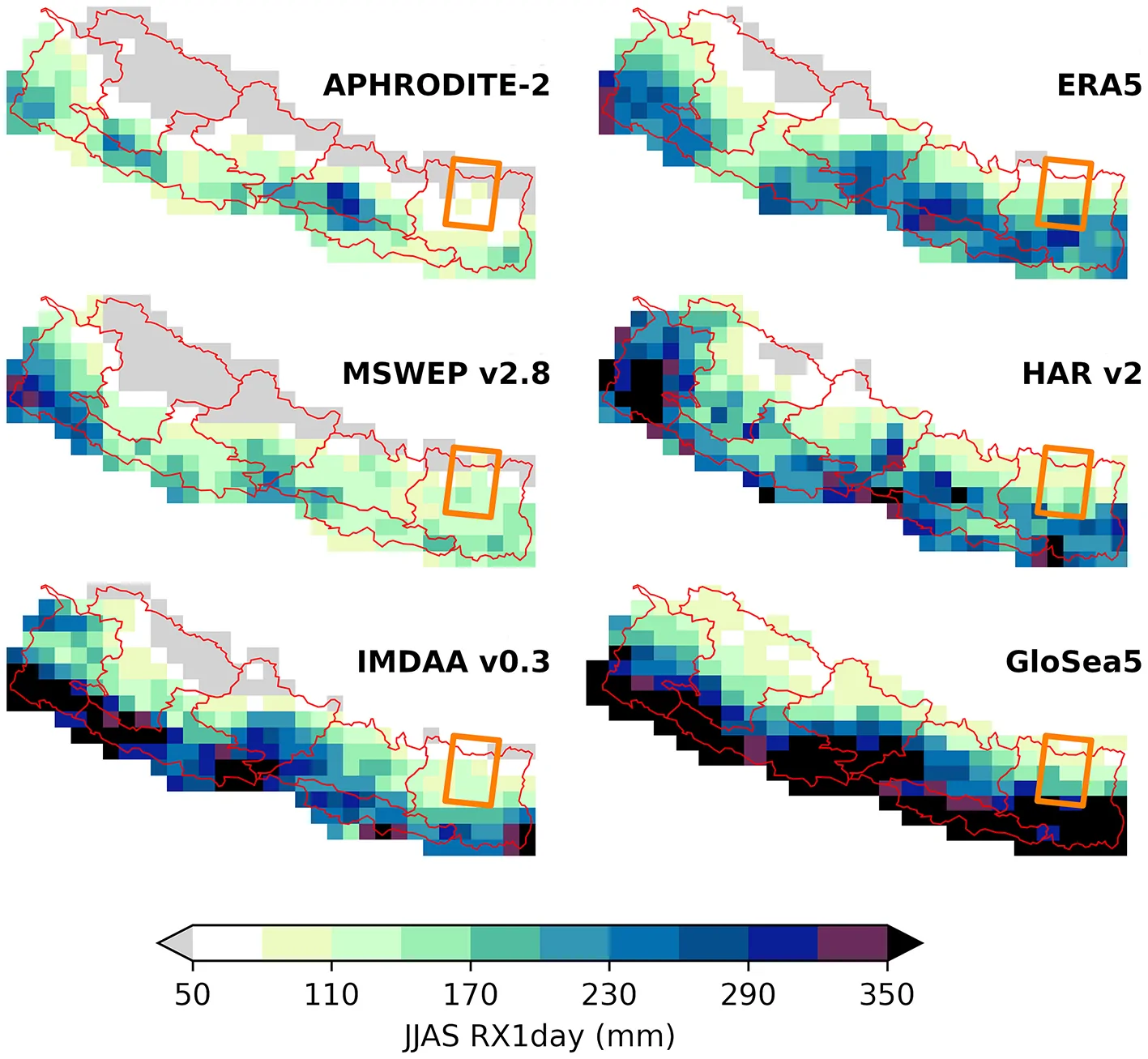
Comparison of 6 rainfall datasets over Nepal. Each dataset has been regridded to a 0.25°x0.25° grid to facilitate a fair comparison of their grid cell estimates of the 2000–2015 mean daily maximum accumulation (RX1day) during June–September (JJAS). The location of the Arun river basin (within Nepal) is marked by the orange rectangle (after ICIMOD, 2010).

There are many sources of precipitation data over Nepal (e.g., Ceglar et al., 2017). For the purposes of demonstration, we examine five open-access datasets and one seasonal forecast system (summarized in Table 1), but in principle the method is agnostic to the number of datasets used. The differences in their estimates of the mean monsoon-total precipitation and monsoon maximum daily-total are presented in Figure 1. Each dataset has been conservatively regridded from their original resolution to a common 0.25°x0.25° grid, and timebound to the commonly shared period of 2000–2015. Even visually, the differences in spatial variability and magnitude of rainfall accumulation are apparent. From a policy makers' perspective: should a new hydropower plant in the Arun river basin (Koshi Pradesh, eastern Nepal, see Figure 1) plan to accommodate a maximum 1-day rainfall accumulation of < 50–110 mm (APHRODITE-2) or >350 mm (GloSea5)?

**Table 1:** Summary of observational datasets used in this study.

**Name**	**Data source**	**URL access**	**References**
Asian Precipitation—Highly-Resolved Observational Data Integration Toward Evaluation (APHRODITE-2) v1901	Rain gauges	http://aphrodite.st.hirosaki-u.ac.jp/index.html	Yatagai et al., 2012
Multi-Source Weighted-Ensemble Precipitation (MSWEP) v2.8	Merged rain gauge estimates with ERA5 and satellite data	http://www.gloh2o.org/mswep/	Beck et al., 2019
High Asia Refined analysis (HAR) v2	Dynamically downscaled reanalysis based on ERA5	https://www.klima.tu-berlin.de/index.php?show=daten_har2	Wang et al., 2021
Indian Monsoon Data Assimilation and Analysis (IMDAA) v0.3	Reanalysis	https://rds.ncmrwf.gov.in/	Rani et al., 2021
ERA5	Reanalysis	https://doi.org/10.24381/cds.adbb2d47	Hersbach et al., 2020
GloSea5	Ensemble seasonal prediction system	Not openly accessible	MacLachlan et al., 2015

**Figure 2:**
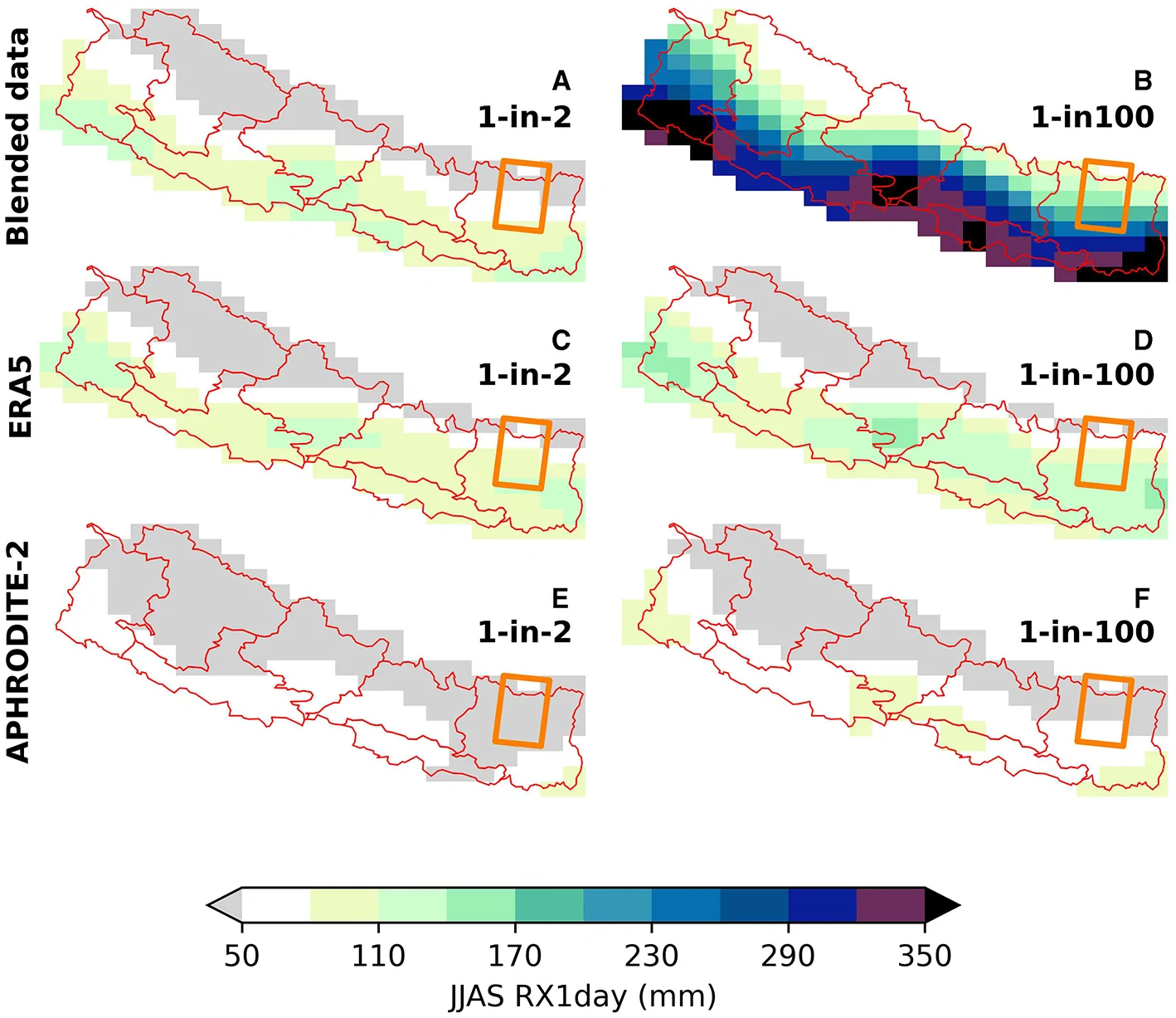
Extreme value estimates of 1-in-2 and 1-in-100 year JJAS RX1day events over Nepal. **(A, B)** Extreme value estimates based on blended data estimate incorporating MSWEP, HAR, IMDAA and GloSea5 data, **(C, D)** ERA5 only and **(E, F)** APHDRODITE-2 only.

The selected source datasets are representative of different methodological approaches to collecting and constructing gridded data. We choose ERA5, a global reanalysis dataset from Hersbach et al. (2020), and APHRODITE-2, a gridded dataset based on *in-situ* rain gauge data from Yatagai et al. (2012), as baseline datasets against which we will compare our blending method. We apply our data blending framework to: MSWEP, a global combined dataset merging rain gauge estimates with ERA5 and satellite data from Beck et al. (2019); HAR v2 from Wang et al. (2021), a regional data set focusing on high mountain Asia, generated by dynamically downscaling ERA5 using the Weather Research and Forecasting model; IMDAA v0.3, a reanalysis dataset from Rani et al. (2021); and GloSea5 from MacLachlan et al. (2015), a seasonal prediction systems based on the HadGEM3 model. These four datasets produce a blended RX1day extreme value estimate. Figure 2 compares the blended result to the baseline datasets for estimates of 1-in-2 and 1-in-100 year RX1day events. RX1day estimates from a single dataset have the potential to significantly misrepresent rainfall accumulation, compared to an estimate derived from a greater number of data sources. This effect is more pronounced for extreme return periods.

**Figure 3:**
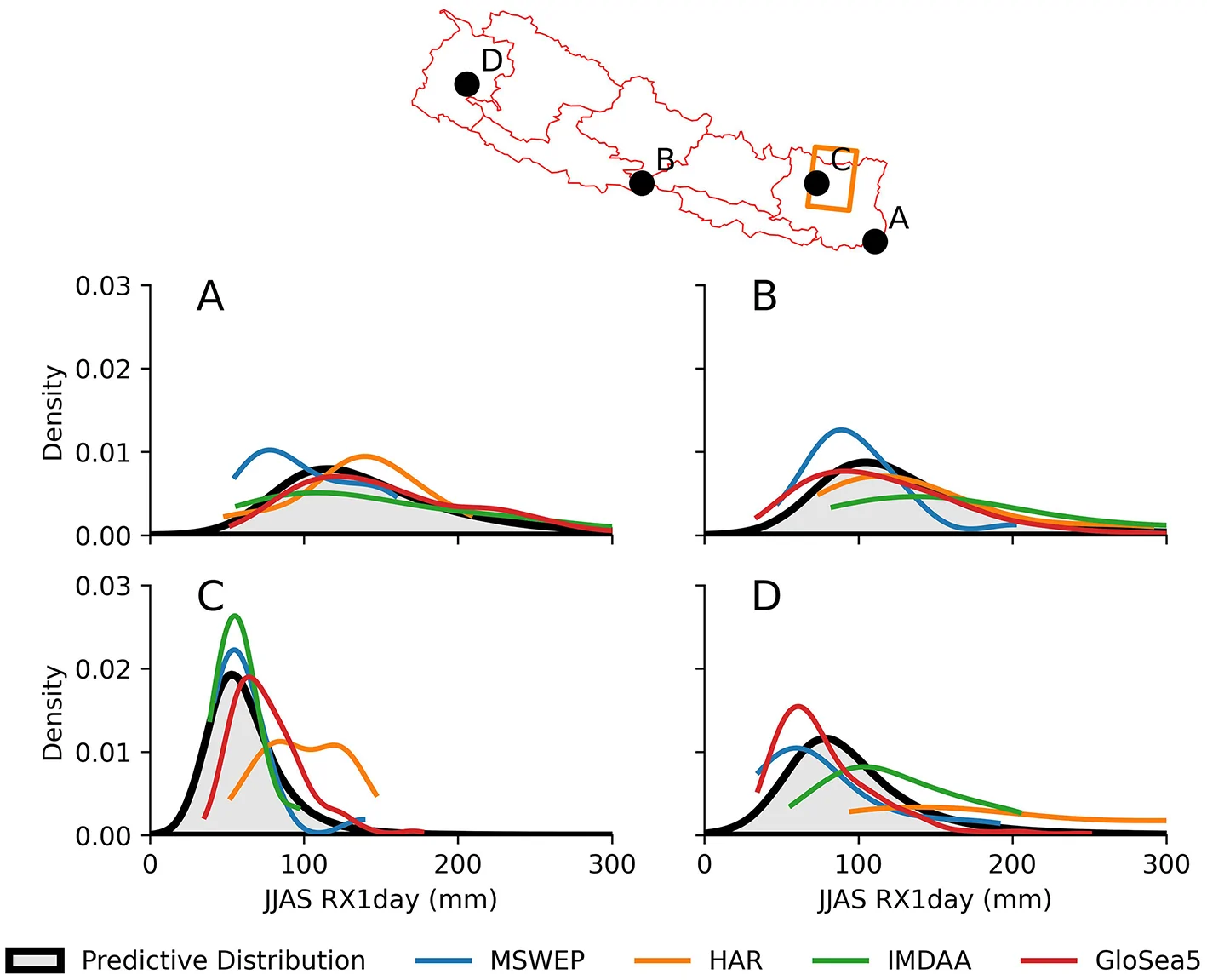
Comparison of the blended model predictive distribution (black, shaded) with input datasets (colored lines) at four grid boxes **(A–D)**. Each panel compares the kernel density estimate (KDE) of the individual datasets, with the blended output. KDE curves are truncated at their data limits. Although **(A–D)** illustrate a single grid box, the predictive distribution is also influenced by surrounding grid boxes.

We also illustrate the interpretability of this method by comparing the predictive distribution from the blended dataset, with the four input datasets (Figure 3). This comparison provides some insight into the data blending, whilst illustrating the consensus (or lack of) in the input datasets. For the blended dataset, the predictive distribution at each location illustrated in Figure 3 is the product of each of the four input data at that location (A–D), but is also influenced by the surrounding values as well. For locations (A) and (C), there is significant variation between the four input datasets, such that the predictive distribution mediates the disparity, supporting the middle-ground. For (B), 3 of 4 datasets support a heavier tailed distribution than MSWEP, but the bulk of the predictive distribution is not as extreme as IMDAA. For (C), HAR is a clear outlier, but with strong agreement between MSWEP and IMDAA which has large influence on the predictive distribution.

## 4. Discussion

Often, ostensibly similar atmospheric datasets show marked differences in their estimate of reality. This is because their reliability is defined by the spatial coverage of surface stations, satellite algorithms, and the data assimilation models that contribute to their creation. Rain gauges provide relatively accurate and trusted measurements of precipitation at point locations, but are unavailable over many sparsely populated and oceanic areas. Satellite observations provide data with a greater degree of homogeneous spatial coverage, but contain non-negligible random errors and biases owing to the indirect nature of the relationship between the measurement from satellite-mounted instrumentation and precipitation at the Earth's surface, inadequate temporal sampling given the satellite's motion in space, and deficiencies in the data processing algorithms needed to amalgamate their observations. Further datasets can be created by incorporating observations into numerical models that use mathematically defined physical processes to generate a synthesized estimate of precipitation across a uniform grid, with spatial homogeneity and temporal continuity.

Where a heuristic measure to identify a single best data source is impossible, and differences in data sources are large, it is difficult to a priori justify the use of a single data source in the decision making process. Approaches such as Bayesian melding (Poole and Raftery, 2000) have been proposed to this end, but with relatively little uptake, possibly due to the underlying complexity of the Bayesian framework, the lack of extension to non-Gaussian variables (such as precipitation) and the challenges associated with scaling this approach to large spatio-temporal datasets. Instead, we propose an approach based on Hierarchical Generalized Additive Models (HGAMs) (Hastie and Tibshirani, 1990; Wood, 2017; Pedersen et al., 2019). This flexible data modeling framework retains the ability to incorporate multiple sources of information and aggregate them into a single summary that is more informative than its constituent parts, but with easier model creation, good computational scalability and an ability to apply the method to non-Gaussian fields. The results of applying a HGAM to the data in Figure 1 are shown in Figure 2.

It is important that a data blending method retains an ability to discern how each separate dataset influences the blended output, such as in Figure 3 (i.e. it is interpretable), otherwise its utility for transparent decision making is no better than an arbitrary a priori judgment of an individual dataset. Uncertainty estimation is also a key part of interpretability: to understand where there is agreement between data sources and appreciate the overall uncertainty of the resulting blended outcome. Crucially, the framework provided by GAMs allows the posterior predictive distribution to include all associated uncertainty (Wood, 2017). Probabilistic approaches have added value for decision making because they can be used in conjunction with decision theory to make rigorously repeatable decisions and quantifying the value (or utility) of the blended data.

Decision making requires quantification of risk and an appreciation of uncertainty. Whilst data only represents part of the decision-making landscape, data science for decision-making should focus on methods that provide interpretable and transparent frameworks for reproducible data blending. The proliferation of atmospheric datasets shouldn't be part of the decision makers' problem but, via data science, provide an approachable foundation on which to make informed decisions.

## Data availability statement

The raw data supporting the conclusions of this article will be made available by the authors, without undue reservation.

## Author contributions

Conceptualization, methodology, writing—original draft, and writing—review and editing: HS and TE. Investigation and visualization: HS. All authors contributed to the article and approved the submitted version.
