# Supplementary material for: Proliferation of atmospheric datasets can hinder policy making: a data blending technique offers a solution
Source: Front Big Data. 2023 Aug 8;6:1198097. doi: 10.3389/fdata.2023.1198097 (PMC10446837; doi:10.3389/fdata.2023.1198097)
Supplement: Supplementary file 1 [file Data_Sheet_1.docx]

Supplementary Material

Proliferation of atmospheric datasets can hinder policy making: a data blending technique offers a solution

**Hamish Steptoe*^1^ & Theo Economou^2^**

^1^Met Office; FitzRoy Road, Exeter, EX13PB, UK

^2^Climate and Atmosphere Research Centre - CARE-C, The Cyprus Institute, Nicosia, Cyprus

*** Correspondence:**Hamish Steptoe ([hamish.steptoe@metoffice.gov.uk)](mailto:hamish.steptoe@metoffice.gov.uk))

# R code

R code used to fit the model in mgcv v1.8-36 is as follows:

fmla.gam <- list(prcp ~ s(cyear, bs='cs', k=3) + te(lon, lat, k = c(12, 9), bs = "ds", m = c(2, 0.5)) + t2(lon, lat, model, k = c(12, 9, 4), bs = c("ds", "ds", "re"), m = c(1, 0.5), full=TRUE),
~ s(cyear, bs='cs', k=3) + te(lon, lat, k = c(12, 9), bs = "ds", m = c(2, 0.5)) + t2(lon, lat, model, k = c(12, 9, 4), bs = c("ds", "ds", "re"), m = c(1, 0.5), full=TRUE),
~ s(model, k=4, bs='re'))

model <- gam(fmla.gam, data=data, method = "REML", optimizer="efs", family=gevlss, weights = data$weights))

where data is a dataframe containing the individual model data.
